# Gastrointestinal stromal tumor: clinicopathological characteristics and pathologic prognostic analysis

**DOI:** 10.1186/s12957-018-1532-1

**Published:** 2018-12-03

**Authors:** Chayanit Jumniensuk, Mongkon Charoenpitakchai

**Affiliations:** 1 Department of Pathology, Army Institute of Pathology, Bangkok, Thailand; 2 Department of Pathology, Phramongkutklao College of Medicine, Bangkok, Thailand

**Keywords:** Gastrointestinal stromal tumor, GIST

## Abstract

**Objective:**

This study aimed to understand clinicopathological characteristics of gastrointestinal stromal tumors (GISTs) and correlation between pathologic features and clinical outcome.

**Methods:**

We used 76 cases diagnosed as primary GISTs during January 2007 to July 2017 at Army Institute of Pathology, Thailand. Clinical, survival, and pathological data were collected and analyzed.

**Results:**

Ages of the patients ranged from 15 to 88 years (M:F = 1:1). The most common presentation was gastrointestinal bleeding (39.7%). The most common site was the stomach (64.5%). Tumor size ranged from 0.6 to 25.5 cm (average 8.78 cm). Histologic types were spindle cell type (75%), mixed spindled-epithelioid type (17.1%), and epithelioid type (7.9%). The majority of histologic subtype was diffuse hypercellularity (67.1%). Tumor necrosis was found in 38.1% and 80% showed low mitotic counts. Most gastrointestinal stromal tumors (27.6%) are low-risk category according to Miettinen and Lasota’s algorithm. Metastasis was found in 27.7%, mostly occurs within 2 years, and is correlated with tumor size > 10 cm (*P* = 0.023), non-spindle cell histologic type (*P* = 0.027), mitotic count > 5/5mm^2^ (*P* = 0.000), myxoid change (*P* = 0.011), and mucosal invasion (*P* = 0.002). Recurrence was found in 8.1%, mostly occurs within 7 years, and correlated with myxoid change (*P* = 0.045).

**Conclusion:**

We found that most of GISTs show spindle cell type and low-risk category. Metastasis was correlated with tumor size > 10 cm, non-spindle cell histologic type, mitotic count > 5/5mm^2^, myxoid change, and mucosal invasion. Recurrence was correlated with myxoid change.

## Introduction

Gastrointestinal stromal tumor (GIST) is the most common primary mesenchymal tumor of the gastrointestinal tract, and the clinical behavior ranges from benign to malignant. Before the pathogenesis of GIST was understood, most GISTs were formerly diagnosed as leiomyoblastomas and gastrointestinal autonomic nerve tumors (GANTs). GIST arises from interstitial cells of Cajal (ICC) and generally characterized to be immunohistochemically positive for KIT (CD117) and contains *KIT-* or PDGFRA-activating mutations [1, 4]. Based on the results of population-based studies in Iceland and Sweden, the incidence of GIST is approximately 11–14.5 per 100,000 per year [1]. After the accuracy in diagnosis of GIST is increased, the annual incidence of GISTs in the USA rises from 300 to 500/year to 5000 to 6000/year [5]. Etiology of GISTs mostly is sporadic, while approximately 10% are associated with syndromes such as succinate dehydrogenase complex deficiencies, Carney triad, Carney Stratakis syndrome, neurofibromatosis type 1 (NF1), and PDGFRA-activating germline mutations [1, 4].

GISTs typically occur in older adults, median age about 60–65 years, and equal in men and women [1]. The most common location of GISTs is the stomach, which accounts for 60% [1, 6], followed by jejunum and ileum approximately 30%, duodenum 5% [2], and colorectum 5% [3, 4], and minority of cases occur in esophagus, appendix, gallbladder, mesentery, omentum, and retroperitoneum [6]. Presentation of GIST is non-specific and varies from abdominal pain, gastric ulcer, gastrointestinal bleeding, and incidental finding from imaging studies [1–4]. Clinical behaviors of GISTs range from benign to malignant. Malignant GIST of the stomach accounts for 25% of gastric GISTs [1] while malignant GIST of the small intestine accounts for 35–40% of small intestinal GISTs [2].

Histologic features of GISTs compose of spindled, epithelioid, or mixed spindled and epithelioid type. The most common is spindle cell type. Nuclear pleomorphism can be seen especially in epithelioid cell type. Furthermore, spindled GISTs can be divided into histologic subtype: sclerosing, palisaded-vacuolated, diffuse hypercellularity, and sarcomatoid features with significant nuclear atypia and mitotic activity. Histologic subtype of epithelioid GISTs consists of sclerosing, discohesive, diffuse hypercellularity, pseudopapillary pattern, and sarcomatous morphology with significant atypia and mitotic activity [1, 4]. SDH-deficient GISTs usually show epithelioid morphology, multinodular with plexiform mural involvement, lymphovascular permeation, and lymph node metastasis [4].

Most GISTs show immunoreactivity to CD117; approximately 5% of GISTs show CD117 negative especially in GISTs with PDGFRA mutation [4]. Prognostic factors of GISTs depend on tumor size and mitotic activity per 5 mm^2^ [1].

We retrospectively studied 76 cases of GISTs aimed to understand the clinical, histomorphological, and immunohistochemical characteristics and pathologic prognostic analysis of GISTs.

## Materials and methods

Patients who were diagnosed with GISTs between 2007 and 2017 were identified by reviewing the pathology department archives at the Army Institute of Pathology. Seventy-six cases were identified with hematoxylin and eosin (H&E) slides and CD117 immunostain slides available for revision. This study was approved by the Institutional Review Board, Royal Thai Army Medical Department.

Clinical data such as age, gender, tumor location, tumor size, signs and symptoms, surgical treatment, medical treatment, and follow-up data were retrospectively reviewed. Tumor size was evaluated according to the maximum tumor dimension. Surgical resection margins were classified as R0–R2 according to the Union for International Cancer Control (UICC) International Union Against Cancer. R0 resection was defined as complete resection of the localized tumors, R1 resection was defined as microscopic residual tumor, and R2 resection was defined as grossly residual tumors. Recurrence was defined as the appearance of macroscopic tumor at the site of original resection. Metastasis was defined as the appearance of tumor distant to the site of the resection.

Hematoxylin and eosin (H&E) slides from each case were re-examined by two pathologists, separately. The following parameters were recorded: histological type (spindled, epithelioid, or mixed), histological subtype (palisaded-vacuolated type, sclerosing type, diffuse hypercellularity, sarcomatoid features with significant nuclear atypia and mitotic activity, discohesive, pseudopapillary), microscopic arrangement (interlacing-bundled, solid), cellularity (high, intermediate, low), nuclear atypia (low, moderate, high), cellular pleomorphism (low, moderate, high), nucleoli, cytoplasmic appearance (eosinophilic, clear, mixed), intranuclear inclusion, skeinoid fibers, rhabdoid appearance, myxoid stroma, sclerotic stroma, hyalinized blood vessels, peritumoral lymphoid cuff, mucosal invasion, invasion of smooth muscle, calcification, hemorrhage, necrosis, and lymph node metastasis. Histological cell type was categorized as spindled (> 75% of the tumor), epithelioid (> 75% of the tumor), or mixed cell type (both spindle and epithelioid at least 25% of the tumor). Mucosal invasion was defined as the infiltration of tumor cells across the muscularis mucosae and extending into the lamina propria. Mitotic counts were determined by evaluating the most cellular section of the neoplasm and counting 5 mm^2^ using an Olympus CX-23 microscope with a × 40 objective and an × 10 ocular (0.196 mm^2^). Immunohistochemical reactivity to the following antibodies was noted: CD117, CD34, smooth muscle actin (SMA), S-100, Desmin, and DOG1.

All cases were stratified into risk groups based on location, size of the tumor, and mitotic counts according to Miettinen and Lasota’s algorithm [24] into none, very low, low, intermediate, and high-risk categories.

### Statistical analysis

Pearson chi-square and Fisher’s exact test were used to assess the association of categorical variables. The Kaplan-Meier method was used to assess recurrence-free survival and metastasis-free survival. The recurrence-free survival was calculated as the time from the date of diagnosis to the date of last follow-up or the date of recurrence. The metastasis-free survival was calculated as the time from the date of diagnosis to the date of last follow-up or the date of metastasis.

## Results

### Demographic data

The study group comprises of 38 men (50%) and 38 women (50%). Ages of the patients ranged from 15 to 88 years, mean age of 61.18 ± 14.13 years, and more than half occur in age > 60 years (41 cases, 53.9%). Among the study group, 8 patients had underlying tumors consisting of invasive mammary carcinoma in 2 patients, gastric adenocarcinoma in 2 patients, adenocarcinoma of sigmoid in 1 patient, clear cell renal cell carcinoma in 1 patient, dermatofibrosarcoma protuberans in 1 patient, and serous cystadenoma of pancreas in 1 patient.

### Clinical data

The most common presentation was gastrointestinal bleeding in 29 patients (39.7%), followed by abdominal pain in 23 patients (31.5%), incidental finding in 11 patients (15.1%), abdominal mass in 10 patients (13.7%), anemic symptoms in 4 patients (5.5%), gastrointestinal obstruction in 2 patients (2.7%), and intussusceptions in 2 patients (2.7%).

The most common location was the stomach in 49 patients (64.5%), followed by the small intestine, rectum, peritoneum, pancreas, and urinary bladder (Table 1).

**Table 1:** Locations of tumors

Locations	Number (%)
Stomach	49 (64.5%)
Duodenum	3 (3.9%)
Jejunum	11 (14.5%)
Ileum	1 (1.3%)
Small intestine, unspecified	4 (5.3%)
Rectum	3 (3.9%)
Pancreas	1 (1.3%)
Urinary bladder	1 (1.3%)
Mesentery	2 (2.6%)
Peritoneum	1 (1.3%)

Tumor sizes ranged from 0.6 to 25.5 cm with a mean size of 8.78 ± 5.6 cm. Median size was 6.8 cm. Most of the tumors were > 5–10 cm (31 cases, 40.8%) (Table 2).

**Table 2:** Size of GISTs

Tumor size, cm	Number (%)
≤ 2	2 (2.6)
> 2–≤ 5	21 (27.6)
> 5–≤ 10	31 (40.8)
> 10–≤ 15	11 (14.5)
> 15–≤ 20	8 (10.5)
> 20	3 (3.9)

Sixty-two patients received surgical treatment (81.6%); of these, 60 patients (78.9%) had complete resection of the localized tumors (R0) and 2 patients (2.6%) had microscopic residual tumor (R1).

### Pathologic data

**Fig. 1:**
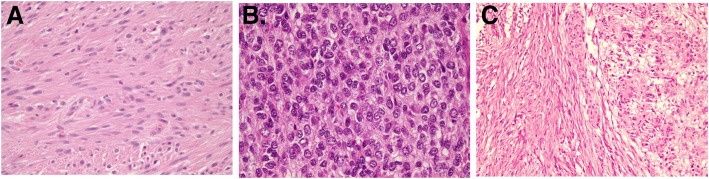
Histologic type of GIST. **a** Spindled cell type (H&E stain, × 400). **b** Epithelioid type (H&E stain, × 400). **c** Mixed spindled-epithelioid type (H&E stain, × 100)

**Fig. 2:**
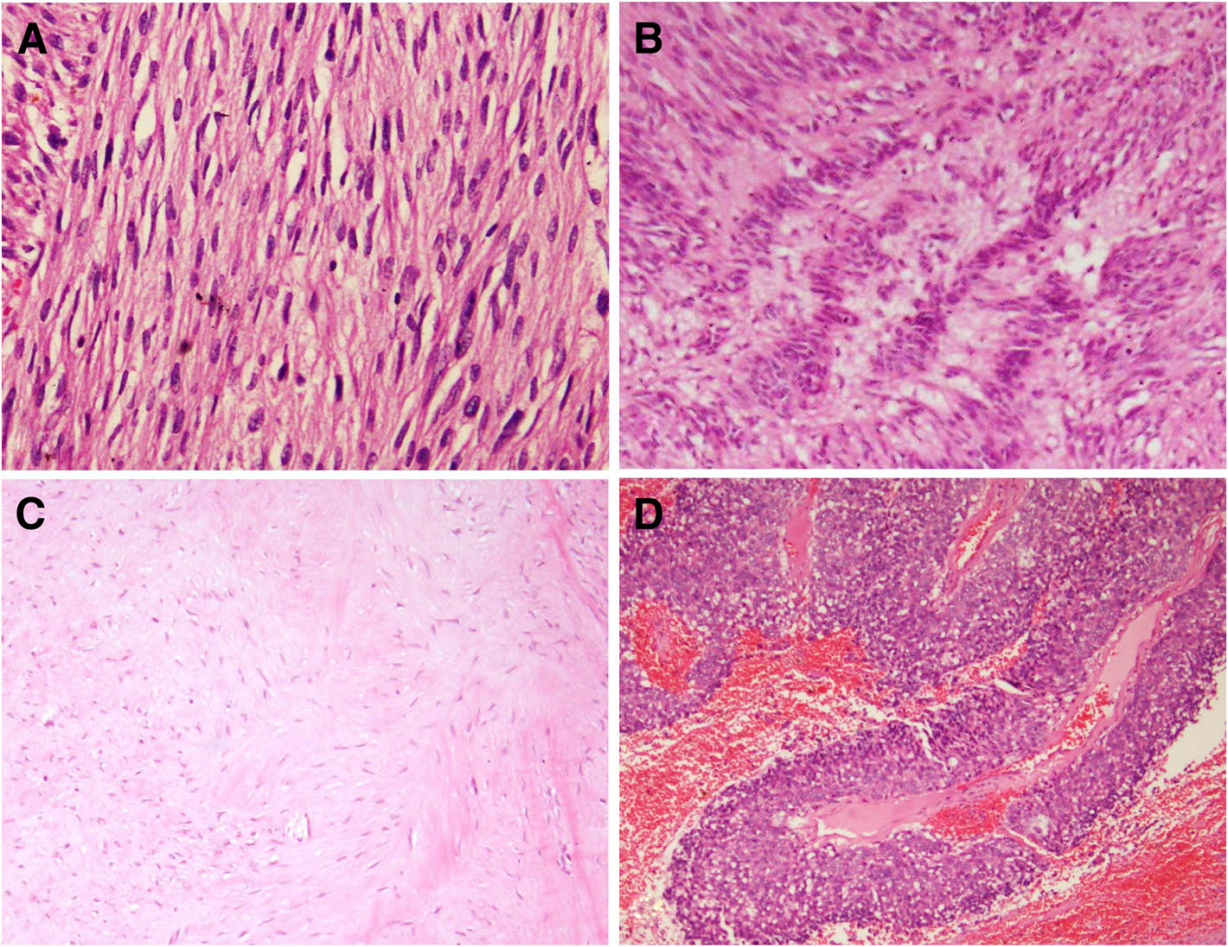
Histologic subtype of GIST. **a** Diffuse hypercellularity subtype contains densely packed diffuse sheets of neoplastic cells (H&E stain, × 100). **b** Palisade-vacuolized subtype shows nuclear palisading (H&E stain, × 100). **c** Sclerosing subtype with extensive collagen deposition (H&E stain, × 40). **d** Pseudopapillary subtype in epithelioid GIST (H&E stain, × 40)

Most of the tumors are unifocal (70 cases, 92.1%) while multifocal is found in 4 cases (5.3%) and 2 cases with unknown status. In this study, the majority of histologic type was spindled cell type, accounting for 57 cases (75%), followed by mixed spindled and epithelioid type in 13 cases (17.1%) and epithelioid type in 6 cases (7.9%) (Fig. 1). The overall histologic subtypes were diffuse hypercellularity in 51 (67.1%), palisade-vacuolated type in 21 (27.6%), sclerosing subtype in 2 (2.6%), and pseudopapillary subtype in 1 (1.3%) (Fig. 2).

The spindled cell tumors were subclassified as diffuse hypercellularity subtype (38 cases, 66.6%), palisade-vacuolated subtype (18 cases, 31.6%), and sclerosing subtype (1 case, 1.8%). Epithelioid GISTs were histologically subclassified as diffuse hypercellularity subtype (5 cases, 83.3%) and pseudopapillary subtype (1 case, 16.7%). The mixed spindled-epithelioid histology was classified as diffuse hypercellularity (9 cases, 69.2%), palisade-vacuolated (3 cases, 23.1%), and sclerosing (1 case, 7.7%) (Table 3).

**Table 3:** Pathologic type and subtype of gastrointestinal stromal tumors

Pathologic type	Number (%)
Spindled cell type	57 (75%)
Diffuse hypercellularity subtype	38 (66.6%)
Palisade-vacuolated subtype	18 (31.6%)
Sclerosing subtype	1 (1.8%)
Epithelioid cell type	6 (7.9%)
Diffuse hypercellularity subtype	5 (83.3%)
Pseudopapillary subtype	1 (16.7%)
Mixed spindled and epithelioid cell type	13 (17.1%)
Diffuse hypercellularity subtype	9 (69.2%)
Palisade-vacuolated subtype	3 (23.1%)
Sclerosing subtype	1 (7.7%)

Microscopic arrangement of the tumor cells revealed solid pattern in 41 cases (53.9%) and interlacing bundle in 34 cases (44.7%). Thirty-nine cases (51.3%) had high cellularity, whereas 37 cases (48.7%) had moderate to low cellularity. Most of the tumors were low-grade nuclear atypia (62 cases, 81.6%) and mild nuclear pleomorphism (56 cases, 73.7%). Nucleoli showed indistinct in most cases (70 cases, 92.1%). Cytoplasm revealed mixed clear and eosinophilic cytoplasm in 53.9%, while 46.1% of the cases were pure eosinophilic cytoplasm and none of them showed pure clear cytoplasm. Nuclear inclusion was found in 7 cases (11.7%). Skeinoid fibers were found in 6 cases (10.2%). Rhabdoid feature was found in 4 cases (6.8%). Intratumoral lymphocytic infiltrations were found in 3 cases (5.1%). Peritumoral lymphocytic aggregation or lymphoid cuff was noted in 7 cases (11.9%). Hemosiderophages were found in 38 cases (60.3%). Myxoid change was found in 4 cases (6.8%).

**Fig. 3:**
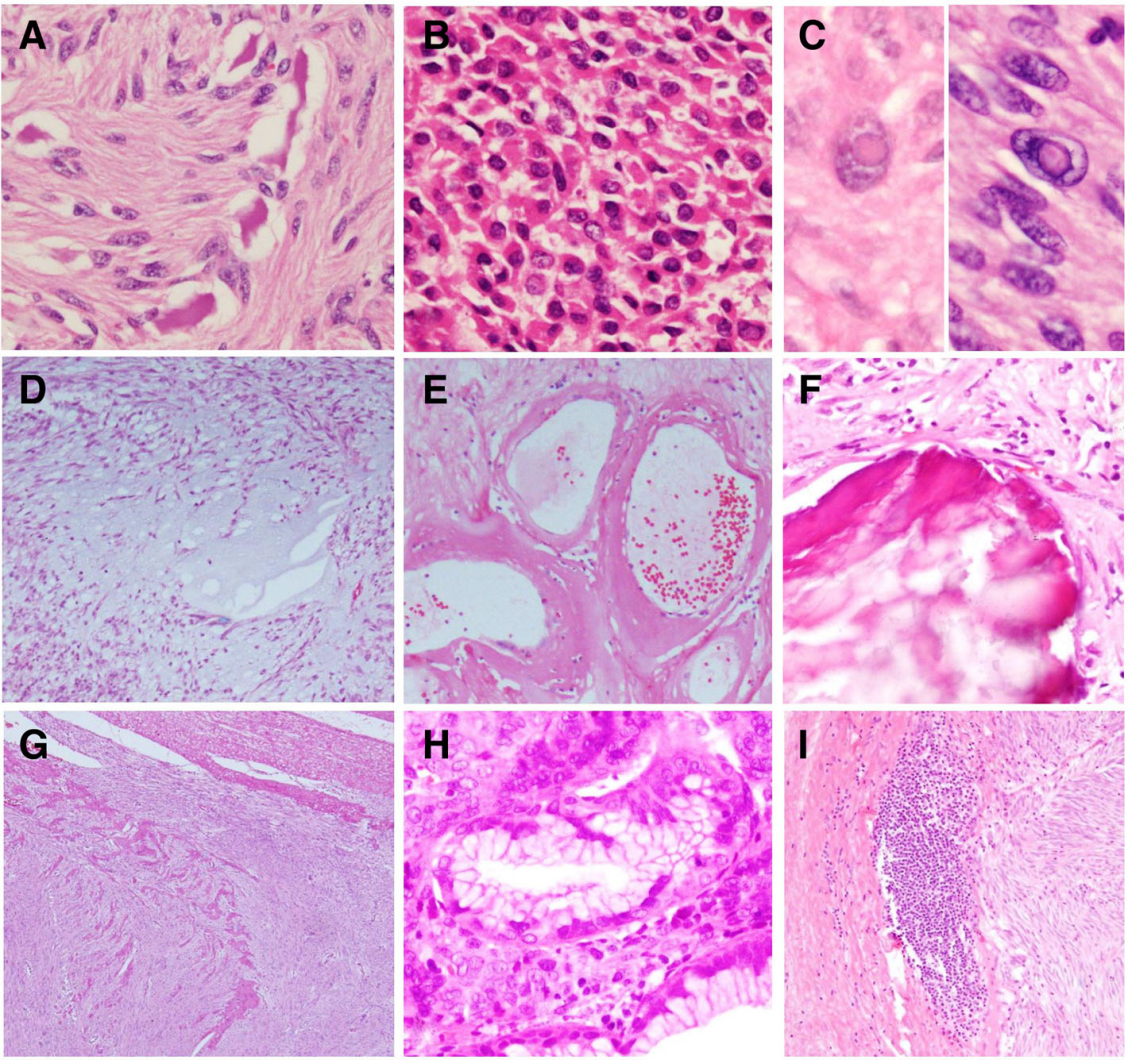
Other features of GIST. **a** Skeinoid fibers. (H&E stain, × 400). **b** Rhabdoid features (H&E stain, × 400). **c** Intranuclear inclusion (H&E stain, × 600). **d** Myxoid changes (H&E stain, × 100). **e** Hyalinized blood vessels (H&E stain, × 400). **f** Calcification (H&E stain, × 100). **g** Muscular invasion (H&E stain, × 40). **h** Mucosal invasion (H&E stain, × 400). **i** Peritumoral lymphocytic aggregation (H&E stain, × 100)

Sclerotic stroma was presented in 4 cases (6.8%). Hyalinized blood vessels were found in 3 cases (5.1%). Calcification was observed in 7 cases (11.7%). Mucosal invasion was reported in 18 cases (29%), whereas muscular invasion was reported in 36 cases (60%). One case showed invasion to adjacent organ (gastric GIST invade esophagus). Coagulative necrosis was found in 24 cases (38.1%) (Table 4 and Fig. 3).

**Table 4:** Histomorphological characteristics of GISTs

Features	Recurrence, *n* (%)^a^			Metastasis, *n* (%)^a^		
	Yes	No	*P* value (*P* < 0.05)	Yes	No	*P* value (*P* < 0.05)
Location			1.000			0.305
Gastrointestinal	5 (100.0)	54 (94.7)		16 (88.9)	45 (95.7)	
Extra-gastrointestinal	0 (0.0)	3 (5.3)		2 (11.1)	2 (4.3)	
Tumor size			0.122			0.023
≤ 10 cm	2 (40.0)	43 (75.4)		9 (50.0)	37 (78.8)	
> 10 cm	3 (60.0)	14 (24.6)		9 (50.0)	10 (21.3)	
Tumor focalty			1.000			1.000
Unifocal	5 (100.0)	52 (94.5)		16 (94.1)	44 (95.7)	
Multifocal	0 (0.0)	3 (5.5)		1 (5.9)	2 (4.3)	
Histomorphological type			0.622			0.027
Spindled cell type	3 (60.0)	41 (71.9)		9 (50.0)	38 (80.9)	
Epithelioid type and mixed type	2 (40.0)	16 (28.1)		9 (50.0)	9 (19.1)	
Histomorphological subtype			1.000			0.114
Diffuse hypercellularity subtype	3 (75.0)	39 (70.9)		14 (87.5)	29 (64.4)	
Palisade-vacuolated subtype	1 (25.0)	16 (29.1)		2 (12.5)	16 (35.6)	
Mitotic count			0.099			0.000
≤ 5/5 mm^2^	2 (40.0)	42 (77.8)		4 (28.6)	41 (87.2)	
> 5/5 mm^2^	3 (60.0)	12 (22.2)		10 (71.4)	6 (12.8)	
Microscopic arrangement			0.376			0.05
Solid	4 (80.0)	31 (54.4)		13 (76.5)	23 (48.9)	
Interlacing-bundled	1 (20.0)	26 (45.6)		4 (23.5)	24 (51.1)	
Cellularity			1.000			0.257
High	3 (60.0)	32 (56.1)		12 (66.7)	24 (51.1)	
Moderate-low	2 (40.0)	25 (43.9)		6 (33.3)	23 (48.9)	
Nuclear atypia			0.855			0.329
High	0 (0.0)	3 (5.3)		1 (5.6)	2 (4.3)	
Intermediate	1 (20.0)	9 (15.8)		5 (27.8)	6 (12.8)	
Low	4 (80.0)	45 (78.9)		12 (66.7)	39 (83.0)	
Cytoplasmic appearance			0.641			0.098
Eosinophilic	3 (60.0)	23 (40.4)		11 (61.1)	18 (38.3)	
Mixed	2 (40.0)	34 (59.6)		7 (38.9)	29 (61.7)	
Skeinoid fibers	1 (20.0)	3 (6.7)	0.353	2 (25.0)	2 (4.8)	0.115
Rhabdoid appearance	0 (0.0)	4 (8.9)	1.000	1 (12.5)	3 (7.1)	0.514
Myxoid change	2 (40.0)	2 (4.4)	0.045	3 (37.5)	1 (2.4)	0.011
Sclerotic stroma	1 (20.0)	3 (6.7)	0.353	1 (12.5)	3 (7.1)	0.514
Intratumoral lymphocytic infiltrations	1 (20.0)	2 (4.4)	0.276	0 (0.0)	3 (7.1)	1.000
Peritumoral lymphocytic aggregation	1 (20.0)	6 (13.3)	0.546	1 (12.5)	6 (14.3)	1.000
Hyalinized blood vessels	1 (20.0)	1 (2.2)	0.192	0 (0.0)	2 (4.8)	1.000
Calcification	0 (0.0)	7 (15.2)	1.000	0 (0.0)	7 (16.3)	0.579
Hemorrhage	4 (80.0)	28 (58.3)	0.637	7 (70.0)	25 (58.1)	0.722
Intranuclear inclusion	0 (0.0)	7 (15.2)	1.000	2 (22.2)	5 (11.9)	0.592
Mucosal invasion	1 (20.0)	13 (27.7)	1.000	7 (70.0)	7 (16.7)	0.002
Muscular invasion	3 (60.0)	28 (60.9)	1.000	6 (75.0)	25 (58.1)	0.456
Coagulative necrosis	4 (80.0)	18 (36.7)	0.146	8 (66.7)	14 (33.3)	0.051

^a^Percentage per column

Mitotic counts range from 0 to 43 per 5 mm^2^ with mean counts of 5.29 ± 8.06 per 5 mm^2^ and median of 2 per 5 mm^2^. Low mitotic counts (≤ 5/5mm^2^) are showed in 55 cases (77.5%) and high mitotic counts (> 5/5mm^2^) are showed in 16 cases (22.5%).

Immunohistochemical studies found that almost all cases were positive for CD117 (98.7%), only one case was CD117 negative, which was positive for DOG1. The others’ positivity were CD34 80%, SMA 20.3%, desmin 2.3%, and DOG1 91.7%. S100 was negative in 71 cases (100%).

The risk stratification was classified according to the Risk Assessment of Primary Gastrointestinal Stromal Tumor (GIST) by Miettinen and Lasota: two cases (2.6%) were none risk, 14 cases (18.4%) were very low risk, 21 cases (27.6%) were low risk, 17 cases (22.4%) were moderate risk, 20 cases (26.3%) were high risk, and 2 cases (2.6%) were insufficient data.

### Follow-up data

Follow-up data were available in 64 patients (84.2%) with the mean of follow-up period of 39.5 ± 29.6 months, range from 0 to 118 months, and median follow-up period of 34.5 months.

Thirty-seven patients (56.9%) were free of diseases, 5 patients (8.1%) had recurrent tumors, and 18 patients (27.7%) had metastatic diseases.

**Fig. 4:**
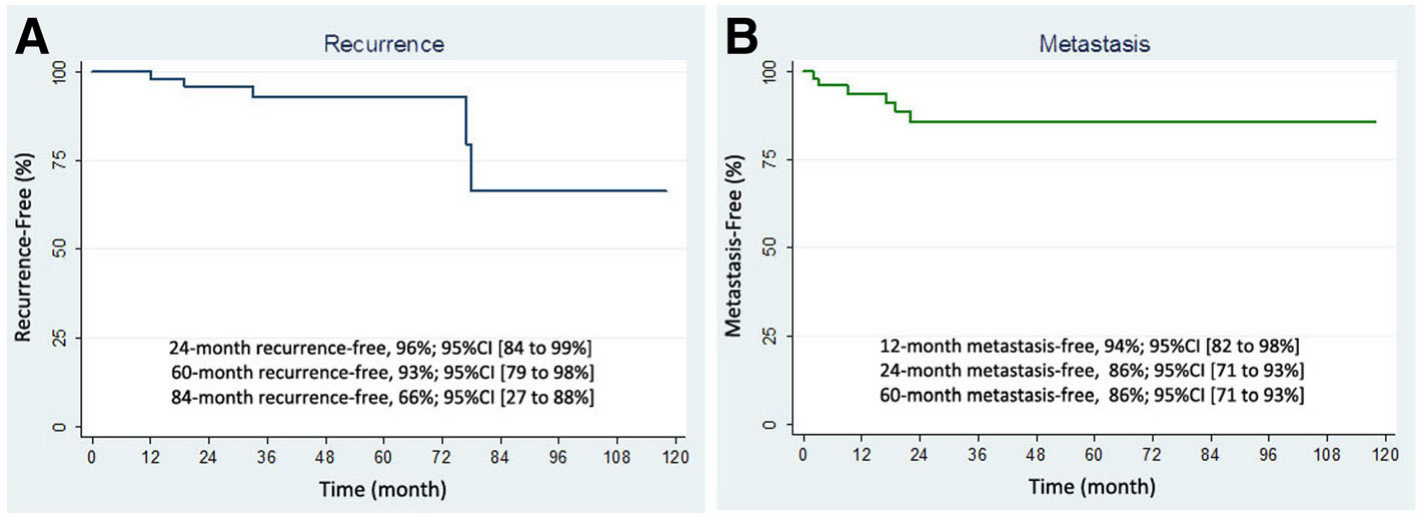
Clinical outcome of patients. **a** Recurrence-free survival. **b** Metastasis-free survival

The period of recurrent disease was found at 12–78 months (median 33 months) after resection of primary tumor, and the mean of recurrent disease after resection was 43.8 ± 31.7 months. For our patients with recurrence tumor, the Kaplan-Meier estimates of the 24-month, 60-month, and 84-month recurrence-free after treatment (Fig. 4) were 96%, 93%, and 66%, respectively. The 95% confidence intervals for these estimates are 84 to 99%, 79 to 98%, and 27 to 88%, respectively.

The period of metastatic disease was found at 0–22 months (median 0 months) after diagnosis of primary tumor with the mean of metastasis occurring at 4 ± 7.4 months. Metastatic diseases were found at diagnosis in 12 patients (18.5%). The most common site of metastasis was the liver (14 cases, 21.5%). The other metastatic sites were the peritoneum (2 cases, 3.1%), omentum (1 case, 1.6%), mesentery (1 case, 1.6%), and bone (1 case, 1.6%). The Kaplan-Meier method of survival analysis estimated that the 12-month metastasis-free survival was 94% (95% CI, 82 to 98%). The 24-month metastasis-free survival was 86% (95% CI, 71 to 93%), and the estimates did not change after 24-month period.

Eight patients died (12.5%). The durations after diagnosis of primary tumor to death range from 0 to 100 months (median 13 months) with the mean of 30 ± 36.9 months. Of these, only one patient had death-related GISTs with massive gastrointestinal bleeding from tumor.

Factor that correlated with recurrence was myxoid change (*P* = 0.045). Factors that correlated with metastasis were tumor size > 10 cm (*P* = 0.023), histologic type (*P* = 0.027), mitotic count > 5/5mm^2^ (*P* = 0.000), myxoid change (*P* = 0.011), and mucosal invasion (*P* = 0.002).

## Discussion

In this study, we identified 76 patients with GISTs. GIST is most occurring in old age group. In our study, the mean age of the patients was 61.18 ± 14.13 years which was supported by the observations of Antonescu et al. [7], Alqusous et al. [12], Din et al. [16], and Kkrishnappa et al. [22]. The minimum age was 15 years which is similar to the study of Antonescu et al. (12 years old). Males (50%) were found equally with females (50%) as in the studies of Lopes et al. [11]. On the contrary, most of the other studies showed males were slightly affected than females including Antonescu et al. [7], Alqusous et al. [12], Din et al. [16], and Tazawa et al. [20]. However, two other series had reported a higher incidence in females compared to males [Eckhard Klieser et al. [10] and Yu Na Kang et al. [21]].

The most common presentation was gastrointestinal bleeding in 29 patients (39.7%), which among this group found the tumor in the stomach in 23 cases (79.3%), jejunum 3 cases (10.3%), and rectum 3 cases (10.3%). This finding may help to be aware that in patients presented with gastrointestinal bleeding while both esophagogastroscope and colonoscope studies were negative, tumor can be located in the small intestine.

The most common location was the stomach in 49 patients (64.5%), followed by the small intestine. This observation was in agreement with that of Ahmed et al. [9], Klieser et al. [10], Lopes et al. [11] Alqusous et al. [12], Vij et al. [13], Yacob et al. [14], Din et al. [16], Li et al. [17], Cao et al. [18], Sui et al. [19], Tazawa et al. [20], Kang et al. [21], and Kkrishnappa et al. [22]. These are in contrast with the two studies of Antonescu et al. [7] and Bhalgami et al. [15] which found slightly more in the small intestine than stomach.

GISTs vary in size, ranging from 0.6 to 25.5 cm. Mean size was 8.78 ± 5.6 cm. The median size of GIST was 6.8 cm. Most of the tumors were > 5–10 cm (31 cases, 40.8%), as in the report by Li et al. [17], while in the USA, Antonescu et al. [7] reported more larger in size, most were ≥ 10 cm with a mean size of 7.8 cm. Tumors larger than 10 cm were found correlated with metastasis, which agrees with the study of Miettinen et al. [23] who found that risk of metastasis increases by tumor size.

Sixty-two patients received surgical treatment (81.6%), most of them (60 cases, 78.9%) were free of tumor at surgical margins (R0). Only 2 patients (2.6%) had microscopic residual cancer at surgical margins (R1), 1 of them had recurrent tumor at 19 months after resection of primary tumor.

In the histopathologic findings, we found that tumors were predominant in spindle cell type (57 cases, 75%), followed by mixed spindled-epithelioid type (13 cases, 17.1%) and epithelioid type (6 cases, 7.9%). These observations were agreed by most of the studies in Asia: Alqusous et al. [12], Vij et al. [13], Bhalgami et al. [15], Li et al. [17], Cao et al. [18], Tazawa et al. [20], and Kkrishnappa et al. [22].

On the contrary, the studies conducted in the USA, Antonescu et al. [7] and Trupiano et al. [8], found that majority were spindle type (84%, 44%), followed by epithelioid cell type (16%, 37%). One of the studies conducted in Pakistan, Din et al. [16], one from China, Sun et al. [19], and one from Korea, Kang et al. [21], also found that majority were spindle type (84.7%, 83%, 88.2%), followed by epithelioid cell type (12.5%, 10%, 9.3%), and the minority were mixed spindled-epithelioid cell type (2.7%, 8%, 2.5%). However, the study of Klieser et al. [10] in Europe found that the majority were spindle type (61.2%), while the epithelioid cell type (19.4%) was equally found with the mixed spindled-epithelioid cell type (19.4%).

Furthermore, Miettinen et al. [1, 4] reported in the World Health Organization (WHO) classification of tumors of the digestive system 2010, and WHO classification of tumors of soft tissue and bone 2013, that most GISTs are spindle cell type, while epithelioid cell type was found approximately 20–25%, and only a small number of cases found mixed spindled-epithelioid histology. These lead to the observation that the distribution of histologic cell type may have some connection with the ethnicity, since the mixed spindled-epithelioid histology was found more common than the epithelioid morphology, which mainly occurs in Asian population.

In our study, the majority of the spindle cell tumors were subclassified predominantly in diffuse hypercellularity subtype (38 cases, 66.6%), followed by the palisade-vacuolated subtype and sclerosing subtype. Similar to the previous study reported by Lopes et al. [11], spindle cell tumors predominantly were diffuse hypercellularity subtype (50.6%), followed by palisading vacuolated subtype (37.3%) and sclerosing subtype (7.4%).

In the previous study reported by Lopes et al. [11], the epithelioid type was histologically subclassified as hypercellular/sarcomatous-like (49.2%), vacuolated (34.4%), pleomorphic (8.2%), sclerosing (6.6%), and organoid (1.6%) subtypes. In contrast with the present study, epithelioid GIST was histologically classified as diffuse hypercellularity subtype (5 cases, 83.3%) and pseudopapillary subtype (1 case, 16.7%). The other subtypes including vacuolated subtype were not identified, may be due to the limited number of cases.

Similar to the epithelioid type, in our study, the mixed spindled-epithelioid histology was classified as diffuse hypercellularity (5 cases, 83.3%) and palisade-vacuolate (1 cases, 16.7%). With the limited number of cases, we cannot identify other subtypes as reported by Lopes et al. [11].

Cellularity of GISTs most were high cellularity (39 cases, 51.3%); this result compared to earlier study showed similarity with the study from Japan, Tazawa et al. [20]. While in the study in India, Vij et al. [13] showed predominant in intermediate cellularity (67, 55.4%).

Skeinoid fibers were found in 6 cases with 5 of 6 were found in the small intestine (1 stomach, 1 ileum, and 4 jejunum). These results similar to the report of Lopes et al. [11] in Brazil showed that skeinoid fibers are found in only 14 tumors (2.7%) in which 12 tumors (85.7%) were located in the small intestine. However, the study in Austria, Klieser et al. [10], found skeinoid fibers in 98 cases (48.8%) which comprises of cases found at the stomach (56 cases) more than cases found at the small bowel (39 cases).

In the present study, coagulative necrosis was found in 24 cases (38.1%) which is similar to the report of Klieser et al. [10] who reported tumor cell necrosis in 70 cases (34.8%) and Alqusous et al. [12] who reported in 17 cases (40.5%). While some studies reported coagulative necrosis less than the present study, Tazawa et al. [20] reported necrosis in 18 cases (31%) and Lopes et al. [11] found necrosis 26.8% of cases.

High mitotic counts per 5 mm^2^ were found correlated with metastasis similar to the report by Miettinen et al. [23] who found that mitotic activities are the most powerful prognosticators integrated with tumor size.

Follow-up data were available in 64 patients (84.2%). The adverse outcome was found in 26 patients out of 64 available data. In this study group, we found that 12 patients (18.5%) had metastatic diseases at first diagnosis. This reported higher rate than the result in the study of Lopes et al. [11] in Brazil, which found first diagnosed as metastatic in 14 cases (2.8%). We also found that there was one case that had metastasis in the bone, in contrast to the data of Miettinen et al. [23] who reported that patterns of metastasis are intra-abdominal dissemination and liver metastases. This should be warrant that there still a chance of metastasis outside the abdomen.

For the recurrent disease, we found that most recurrent tumors occur within 7 years after resection of primary tumor. This finding was agreed by the large study of Miettinen et al. [23] who found that the time intervals from primary tumor to recurrence occur 5 to 33 years indicating that long-term follow-up should be done.

There are some limitations of the present study. First, the present study is a retrospective analysis which lacks systematic prospective. Therefore, completeness of the data is limited. Second, due to the duration of follow-up cases were not long enough, so some recently diagnosed cases of the adverse outcome may not occur.

## Conclusion

We found that most of the GISTs show spindle cell type and low-risk category. Metastasis was correlated with tumor size > 10 cm, non-spindle cell histologic type, mitotic count > 5/5mm^2^, myxoid change, and mucosal invasion. Recurrence was correlated with myxoid change.

## Abbreviations

GANTs: Gastrointestinal autonomic nerve tumors
GIST: Gastrointestinal stromal tumor
H&E: Hematoxylin and eosin
ICC: Interstitial cells of Cajal
SMA: Smooth muscle actin
UICC: Union for International Cancer Control
WHO: World Health Organization

## References

[1] Miettinen M, Fletcher CDM, Kindblom LG, Tsui WMS. Mesenchymal tumors of the stomach. In: Bosman FT, Carneiro F, Hruban RH, Theise ND, editors. WHO classification of tumors of the digestive system. 4th. ed. Lyon: IARC; 2010. p. 74–9.

[2] Miettinen M, Fletcher CDM, Kindblom LG, Tsui WMS, Bosman FT, Carneiro F, Hruban RH, Theise ND (2010). Mesenchymal tumors of the small intestine. WHO classification of tumors of the digestive system.

[3] Miettinen M, Fletcher CDM, Kindblom LG, Tsui WMS, Bosman FT, Carneiro F, Hruban RH, Theise ND (2010). Mesenchymal tumors of the colon and rectum. WHO classification of tumors of the digestive system.

[4] Miettinen MM, Lasota J, Corless CL, Rubin BP, Debiec-Rychter M, Sciot R, Fletcher DM, Bridge JA, Hogendoorn PCW, Mertens F (2013). Gastrointestinal stromal tumors. WHO classification of tumors of soft tissue and bone.

[5] Fletcher CD, Berman JJ, Corless C (2002). Diagnosis of gastrointestinal stromal tumors: a consensus approach. Hum Pathol.

[6] Patil DT, Rubin BP (2011). Gastrointestinal stromal tumor: advances in diagnosis and management. Arch Pathol Lab Med.

[7] Antonescu CR, Sommer G, Sarran L, Tschernyavsky SJ, Riedel E, Woodruff JM (2003). Association of KIT exon 9 mutations with nongastric primary site and aggressive behavior: KIT mutation analysis and clinical correlates of 120 gastrointestinal stromal tumors. Clin Cancer Res.

[8] Trupiano JK, Stewart RE, Misick C, Appelman HD, Goldblum JR (2002). Gastric stromal tumors: a clinicopathologic study of 77 cases with correlation of features with nonaggressive and aggressive clinical behaviors. Am J Surg Pathol.

[9] Ahmed I, Welch NT, Parsons SL (2008). Gastrointestinal stromal tumours (GIST) - 17 years experience from mid trent region (United Kingdom). Eur J Surg Oncol.

[10] Klieser E, Pichelstorfer M, Weyland D, Kemmerling R, Swierczynski S, Dinnewitzer A (2016). Back to the start: evaluation of prognostic markers in gastrointestinal stromal tumors. Molecular and clinical oncology.

[11] Lopes LF, Ojopi EB, Bacchi CE (2008). Gastrointestinal stromal tumor in Brazil: clinicopathology, immunohistochemistry, and molecular genetics of 513 cases. Pathol Int.

[12] Alqusous ST, Rabadi OJ, Omari A, Abbasi N, Haddadin SW, Rawabdeh S (2016). Clinicopathologic spectrum of gastrointestinal stromal tumours; six years experience at King Hussein Medical Center. JRMS.

[13] Vij M, Agrawal V, Kumar A, Pandey R (2010). Gastrointestinal stromal tumors: a clinicopathological and immunohistochemical study of 121 cases. Indian J Gastroenterol.

[14] Yacob M, Inian S, Sudhakar CB (2015). Gastrointestinal stromal tumours: review of 150 cases from a single centre. Indian J Surg.

[15] Bhalgami R, Manish K, Patil P, Mehta S, Mohandas KM (2013). Clinicopathological study of 113 gastrointestinal stromal tumors. Indian J Gastroenterol.

[16] Ud Din N, Ahmad Z, Arshad H, Idrees R, Kayani N (2015). Gastrointestinal stromal tumors: a clinicopathologic and risk stratification study of 255 cases from Pakistan and review of literature. Asian Pac J Cancer Prev.

[17] Li J, Zhang H, Chen Z, Su K (2015). Clinico-pathological characteristics and prognostic factors of gastrointestinal stromal tumors among a Chinese population. Int J Clin Exp Pathol.

[18] Cao H, Zhang Y, Wang M, Shen DP, Sheng ZY, Ni XZ (2010). Prognostic analysis of patients with gastrointestinal stromal tumors: a single unit experience with surgical treatment of primary disease. Chin Med J.

[19] Sui XL, Wang H, Sun XW (2012). Expression of DOG1, CD117 and PDGFRA in gastrointestinal stromal tumors and correlations with clinicopathology. Asian Pac J Cancer Prev.

[20] Tazawa K, Tsukada K, Makuuchi H, Tsutsumi Y (1999). An immunohistochemical and clinicopathological study of gastrointestinal stromal tumors. Pathol Int.

[21] Kang YN, Jung HR, Hwang I (2010). Clinicopathological and immunohistochemical features of gastointestinal stromal tumors. Cancer Res Treat.

[22] Kkrishnappa P, Loh EJ, Mohamad IB, Tata MD, Akhilesh M, Palayan K (2016). Histomorphology and immunohistochemistry of gastrointestinal stromal tumors in a Malaysian population. Asian Pac J Cancer Prev.

[23] Miettinen M, Sobin LH, Lasota J (2005). Gastrointestinal stromal tumors of the stomach: a clinicopathologic, immunohistochemical, and molecular genetic studies of 1765 cases with long-term follow-up. Am J Surg Pathol.

[24] Miettinen M, Sobin LH, Lasota J (2006). Gastrointestinal stromal tumors: review on morphology, molecular pathology, prognosis, and differential diagnosis. Arch Pathol Lab Med.

